# Improving the Quality of Qualitative Interviews Through the Lens of Health Equity

**DOI:** 10.1093/geroni/igaf122.1734

**Published:** 2025-12-31

**Authors:** Weiyu Mao, Jing Wang, Yaguang Zheng, Yaolin Pei, Bei Wu

**Affiliations:** University of Nevada, Reno, Reno, Nevada, United States; University of New Hampshire, Durham, New Hampshire, United States; New York University, New York, New York, United States; The University of Texas at Austin, Austin, Texas, United States; NYU Shanghai, Shanghai, China (People’s Republic)

## Abstract

The contributions of qualitative research to health equity research are increasingly recognized. There remain challenges in incorporating diverse voices of underserved populations into aging research. Although qualitative interviews are valuable for understanding differences in health behaviors and perspectives, they may not adequately capture the complexities of inequities within marginalized communities. To illustrate, we conducted reflexive thematic analysis of three qualitative studies involving Chinese Americans on topics, such as oral health, diabetes technology use, and advanced care planning. Across the studies, we frequently encountered challenges in eliciting detailed responses, and some participants provided only brief or non-informative responses. This issue persisted despite time, efforts, and methodological considerations, such as training and cultural competence of interviewers, pilot testing of instruments, and use of intentional interview techniques. The lack of depth in participant responses presents a risk of overlooking key insights during data collection and analysis, potentially limiting the identification of underlying factors contributing to health inequities. We recognized the need to amplify the voices of individuals from vulnerable backgrounds, such as low socioeconomic status, rural residence, limited language proficiency, acculturation, and health literacy. This highlights the importance of examining how cultural norms, language preferences, personality traits, and communication styles may shape the nature and depth of participants’ responses. This also suggests that inclusive sampling should be enhanced by fostering meaningful engagement with participants. Ensuring data saturation and nuanced understandings of participant diversity requires not only capturing various perspectives but also mitigating interpretative biases that may arise from the underrepresentation of certain subgroups.

